# The biological function of the N6-Methyladenosine reader YTHDC2 and its role in diseases

**DOI:** 10.1186/s12967-024-05293-6

**Published:** 2024-05-24

**Authors:** Xudong Wu, Hui Chen, Kai Li, Hong Zhang, Kai Li, Haoyu Tan

**Affiliations:** 1 Department of Thoracic Surgery, The Third Hospital of Changsha, Changsha, 410015 Hunan People’s Republic of China; 2 Department of Thoracic Surgery, Xiangxi Autonomous Prefecture People’s Hospital, Jishou, 410015 Hunan People’s Republic of China; 3 Department of Cardio-vascular Surgery, The Second Xiangya Hospital of Central South University, Changsha, 410011 Hunan People’s Republic of China

**Keywords:** YTHDC2, N6-methyladenosine, m6A, Biomarkers, Tumor biology

## Abstract

N6-methyladenosine (m6A) stands as the most prevalent modified form of RNA in eukaryotes, pivotal in various biological processes such as regulating RNA stability, translation, and transcription. All members within the YT521-B homology (YTH) gene family are categorized as m6A reading proteins, capable of identifying and binding m6A modifications on RNA, thereby regulating RNA metabolism and functioning across diverse physiological processes. YTH domain-containing 2 (YTHDC2), identified as the latest member of the YTH family, has only recently started to emerge for its biological function. Numerous studies have underscored the significance of YTHDC2 in human physiology, highlighting its involvement in both tumor progression and non-tumor diseases. Consequently, this review aims to further elucidate the pathological mechanisms of YTHDC2 by summarizing its functions and roles in tumors and other diseases, with a particular focus on its downstream molecular targets and signaling pathways.

## Background

**Fig. 1:**
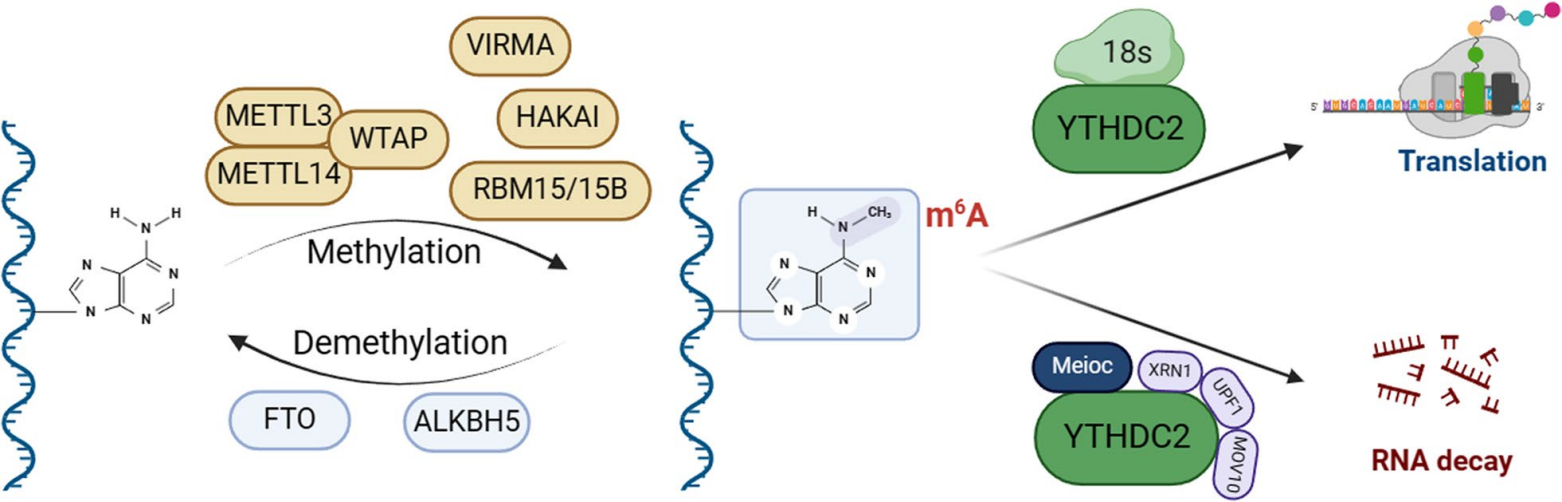
The methylation process of m6A and the role of YTHDC2 in m6A. M6A methylation is involved in a series of enzymes, including m6A methyltransferases and m6A demethylases. YTHDC2 can bind to m6A modified RNA and participate in regulating RNA translation or degradation. Created with BioRender.com

N6-methyladenosine (m6A), the predominant form of RNA modification, plays a crucial role in various cellular biological processes by regulating transcription, translation, stability, processing, splicing, and degradation of target RNA [1, 2]. The regulation of m6A modification primarily involves three key factors: methyltransferases (writers), demethylases (erasers), and m6A RNA-binding proteins (readers) [3]. These proteins are capable of adding, removing, and recognizing m6A modifications on RNA molecules, thereby altering RNA structure and function (Fig. 1) [4]. The addition of m6A methylation is primarily catalyzed by the m6A methyltransferase complex (MTC) [5], with methyltransferase like 3 (METTL3) and methyltransferase like 14 (METTL14 serving as crucial enzymes within this complex [6]. Additionally, cofactors such as Wilms tumor 1-associated protein (WTAP) and RNA-binding motif protein 15 (RBM15) are involved in m6A methylation [7], collectively working to recognize specific adenine bases on RNA and transfer methyl groups from S-adenosylmethionine (SAM) to RNA adenine, thus forming m6A methylation modification [8]. M6A demethylases primarily include fat mass and obesity-related protein (FTO) and alkylation repair homolog protein 5 (ALKBH5), which reduce m6A modification on RNA to unmodified adenine, thereby removing m6A methylation modification [9]. These two m6A demethylases play vital roles in cellular processes by regulating the level of m6A methylation modification, thereby influencing RNA function and stability [10].

M6A reading proteins are a class of proteins that can recognize and bind to m6A RNA modifications, playing an important role in regulating RNA metabolism and function [11, 12]. Among these, the YT521-B homology (YTH) domain family stands as the most conserved and widely distributed m6A reading proteins, playing a significant role in RNA metabolism and regulation [13, 14]. Comprising the YTH domain family are five proteins with YTH domains: YTHDF1, YTHDF2, YTHDF3, YTHDC1, and YTHDC2 [15]. The YTH domain, a common feature among members of this family, typically consists of 130 ~ 140 amino acids and features conserved domain characteristics, including specific amino acid residue sequences and domain folding [16, 17]. The YTH domain typically encompasses multiple β-folding structures and specific RNA-binding pockets capable of directly accommodating modified m6A residues in hydrophobic pockets to exert their effects [18]. Members of the YTH domain family regulate the effects of m6A modification on RNA within cells, thereby influencing gene expression and cellular function [19, 20]. As the most recently discovered member of the YTH domain family, the expression, role, and function of YTHDC2 in the human body have only gradually emerged in recent years [21], enriching our understanding of RNA function regulation by m6A modification. This article will review the research progress of YTHDC2 and explore its potential role in future research.

### Overview of YTHDC2

**Fig. 2:**
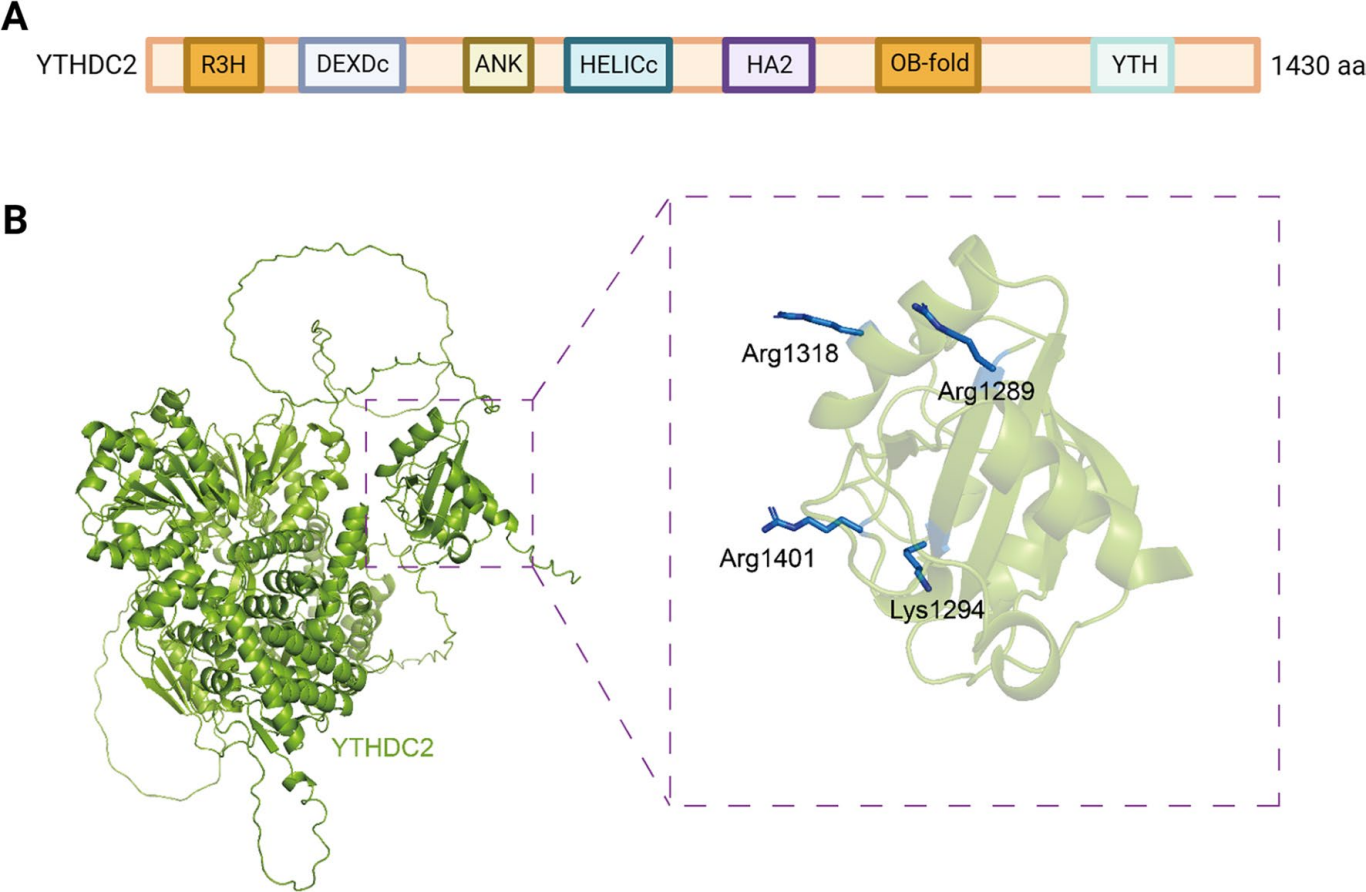
Structure of YTHDC2 domain and its YTH domain. **A**. Schematic of human YTHDC2 domain structure. **B**. Three-dimensional model of the full length human YTHDC2 and its YTH domain. The four basic residues Arg1318, Arg1289, Lys1294, and Arg1401 on the surface of the YTHDC2 YTH domain are crucial for binding to the RNA backbone. The 3D carton was generated with PyMol (http://www.pymol.org)

YTHDC2 encompasses multiple functional domains, with its most notable feature being the YTH domain, enabling recognition and binding of m6A-modified RNA molecules, thus regulating RNA stability, transcription, and translation processes (Fig. 2A) [22, 23]. The YTH domain surface is characterized by four basic residues crucial for RNA backbone binding (Fig. 2B) [14]. Typically, conserved amino acid sequences within the YTH domain form tryptophan cages facilitating interaction with specific RNA bases [24]. However, unlike other proteins with YTH domains, YTHDC2 exhibits a weaker binding affinity with m6A RNA [25]. Research utilizing crosslinking immunoprecipitation (CLIP) to investigate the YTHDC2 binding site in the transcriptome indicated a minimal overlap with the m6A site, differing from other YTH domain proteins [26]. Hence, YTHDC2 may bind to specific m6A sites or employ different binding modes to influence RNA.

YTHDC2 is also known as Probable ATP-dependent RNA helicase YTHDC2, which belongs to the DEAD box helicase family [27], featuring an RNA helicase domain akin to RNA helicases that regulate translation, suggesting a potential role in the promotion of mRNA translation [28]. YTHDC2 has been shown to interact with ribosomal 18s rRNA and simultaneously enhance the RNA translation efficiency of its target gene while reducing its mRNA abundance [21]. Furthermore, YTHDC2 could mediate mRNA degradation by directly interacting with its companion proteins XRN1, UPF1, MOV10 and Meioc [21, 29, 30]. Moreover, YTHDC2 enhanced mRNA translation efficiency by recognizing m6A methylation in the coding region (CDS), with knockdown or silencing of YTHDC2 resulting in a significant reduction in protein synthesis [31].

Distinct from other widely expressed YTH proteins in human tissues, YTHDC2 has been shown to be notably enriched in the testes [21], where it played a crucial role in the development and maturation of germ cells, particularly in sperm development. Of note, YTHDC2 gene knockout mice exhibited defects in spermatogenesis and infertility, without significant developmental defects elsewhere [21]. Male mice displayed markedly smaller testicles compared to littermates, while female mice exhibited similarly smaller ovaries [32]. Depletion of YTHDC2 in mouse testes could slightly upregulate mRNA levels with higher m6A content. YTHDC2 maintained a favorable gene expression program for meiosis by regulating transcriptome levels of m6A-modified germ cell lines [32]. YTHDC2-deficient germ cells could initiate meiosis but underwent premature and abnormal metaphase apoptosis without following a typical meiotic gene expression program [33]. In addition, YTHDC2 participated in chicken embryonic gonadal development sex differentiation process by regulating the expression of gender-related genes, particularly HEMGN and sex determining region Y-box 9 (SOX9) [34]. Additionally, YTHDC2 exhibited a unique subcellular localization, existing in both the nucleus and cytoplasm, unlike YTHDC1 mainly in the nucleus and YTHDF protein primarily in the cytoplasm [34]. These characteristics position YTHDC2 as a crucial regulatory factor in intracellular RNA metabolism, vital for normal cellular function and developmental processes.

### The role of YTHDC2 in tumors

#### The role of YTHDC2 in respiratory system tumors

**Fig. 3:**
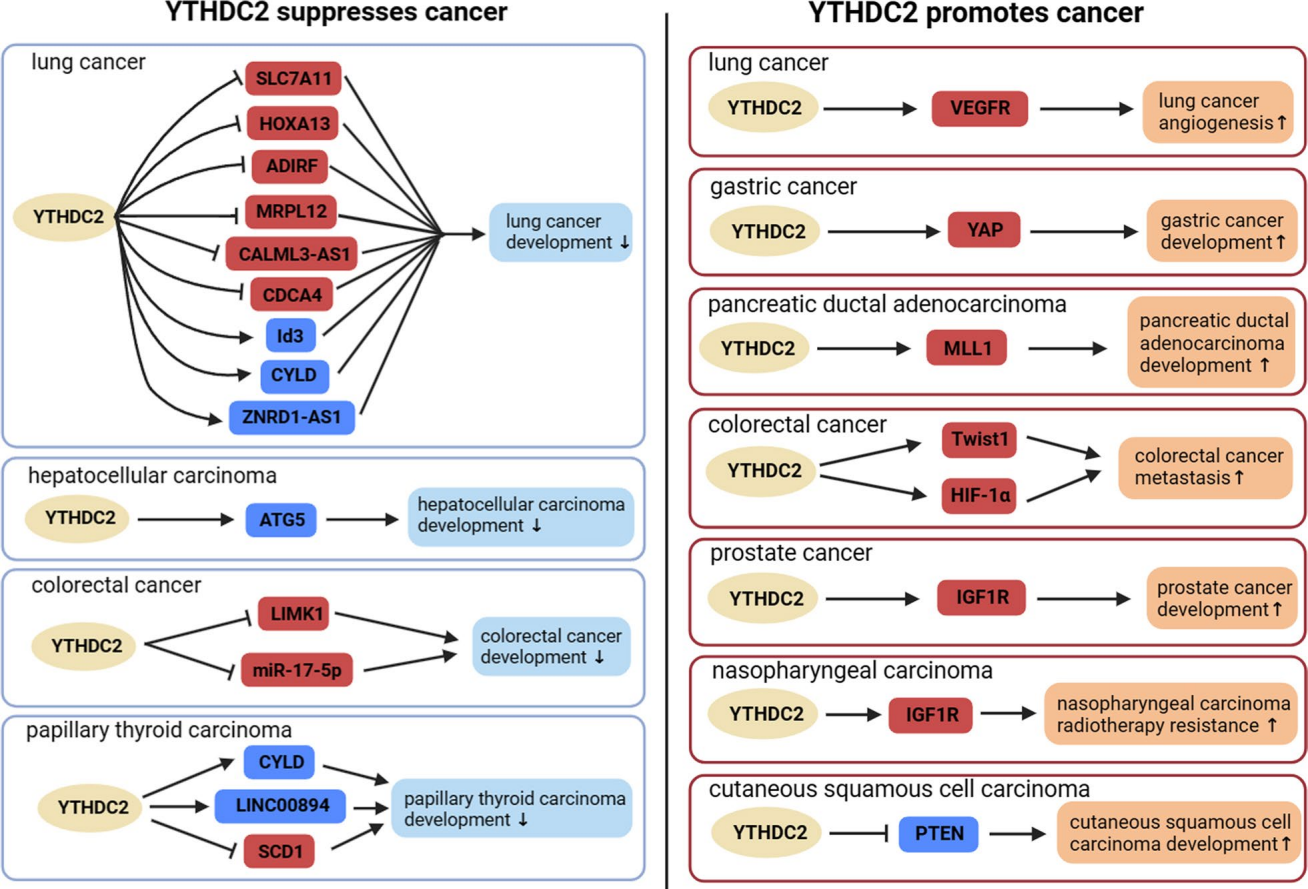
The target gene of YTHDC2 and its role in tumors. YTHDC2 regulates the expression of multiple genes by recognizing m6A modified RNAs and influencing their fate. In tumors, based on the different target genes of YTHDC2, YTHDC2 can exhibit pro-cancer or anti-cancer effects and participate in regulating the occurrence and development of tumors. The red box represents oncogenes, and the blue box represents tumor suppressor genes. Created with BioRender.com

Currently, multiple studies have confirmed the multifaceted role of YTHDC2 in either promoting or inhibiting the occurrence and progression of cancer through diverse mechanisms (Fig. 3). Notably, reduced expression of YTHDC2 has been observed in lung cancer and cigarette smoke-exposed cells, correlating with smoking history, advanced stage, invasion depth, lymph node metastasis, and poor outcomes. Both in vitro and in vivo studies also demonstrated that YTHDC2 enhances the mRNA stability of cylindromatosis (CYLD) in an m6A-dependent manner, thereby inhibiting the activation of the NF-κB signaling pathway through CYLD’s deubiquitination activity, consequently suppressing the proliferation and migration of malignant lung cells [35].

The transportation of cysteine primarily occurs through various transport proteins and transporters, possessing antioxidant properties that protect cells from oxidative damage and ferroptosis [36–38]. Ma et al. found that decreased YTHDC2 expression correlates with poorer prognosis in lung adenocarcinoma (LUAD) [39]. Mechanistically, YTHDC2 was shown to bind directly to the mRNA of m6A-modified solute carrier 7A11 (SLC7A11), promoting its decay and thereby inhibiting cysteine uptake, which blocks downstream antioxidant programs, contributing to its anti-tumor activity. Additionally, YTHDC2 has also been shown to m6A-dependently bind to the 3’-untranslated region (UTR) of Homeobox A13 (HOXA13) mRNA, reducing its RNA stability and inhibiting the transcription of solid carrier 3A2 (SLC3A2), leading to ferroptosis [40].

Furthermore, YTHDC2 interacts with various mRNA targets to regulate LUAD progression. By binding directly to ADIRF mRNA in LUAD, YTHDC2 have been shown to attenuate its expression, while ALKBH5 counteracted this effect, leading to inhibition of LUAD cell proliferation and metastasis [41]. YTHDC2 also targeted m6A-modified mitochondrial ribosomal protein L12 (MRPL12) mRNA, inhibiting LUAD tumor growth and metastasis, while promoting apoptosis, and ultimately suppressing tumor occurrence [42]. Additionally, YTHDC2 inhibited drug resistance in non-small cell lung cancer (NSCLC) by upregulating the expression of the tumor suppressor gene Inhibitor of DNA-binding 3 (ID3) in cisplatin-resistant NSCLC cell lines [43]. Moreover, YTHDC2 modulated LUAD development by regulating the levels of cell division cycle-related protein CDCA4, thereby inhibiting the malignant phenotype and altering the M1/M2 cell ratio in tumor tissue [44].

Beyond mRNA regulation, YTHDC2 have also been shown to inhibit the malignant progression of lung cancer by regulating the levels of long non-coding RNAs (lncRNAs). Wang et al. found that YTHDC2 promoted the stability and levels of lncRNA ZNRD1-AS1, which inhibits lung cancer cell proliferation, migration, and angiogenesis in vitro and in vivo through the miR-942/TNS1 axis, resulting in reduced tumor growth in nude mice [45]. Conversely, YTHDC2 negatively regulated the levels of lncRNA CALML3-AS1 in an m6A modification-dependent manner, leading to upregulation of tumor suppressor gene butyrophilin like 9 (BTNL9) and inhibit NSCLC proliferation, invasion, and migration [46].

However, contrary to previous studies, Zhang et al. reported a pro-cancer effect of YTHDC2 in lung cancer. YTHDC2 was shown to form complexes with eukaryotic translation initiation factor 4 gamma 1 (eIF4GI) and bind to the m6A methylation site of the vascular endothelial growth factor A (VEGFA) 5’UTR, thereby promoting its translation and accelerating lung cancer angiogenesis [47]. These contrasting roles of YTHDC2 in lung cancer underscore its context-dependent functions via different downstream targets, suggesting personalized therapeutic strategies based on individual patient profiles.

#### The role of YTHDC2 in digestive system tumors

YTHDC2 facilitates the proliferation, migration, and invasion of gastric cancer (GC) cells both in vivo and in vitro. It has been shown to promote the translation of the oncogene Yes1 associated transcriptional regulator (YAP) mRNA, and YAP, in turn, directly binds to the YTHDC2 promoter, enhancing YTHDC2 transcription, thus establishing a positive feedback loop that amplifies the pro-cancer effect [48]. In tumors, ferroptosis can lead to iron-dependent cell death, thereby inhibiting tumor growth and metastasis [49]. Li et al. observed a significant association between ferroptosis in hepatocellular carcinoma (HCC) and high levels of m6A modification. YTHDC2 upregulated autophagy related 5 (ATG5) expression post-transcriptionally in an m6A-dependent manner, promoting ferroptosis and inhibiting HCC development [50]. However, contrary to the aforementioned findings, Tanabe et al. discovered that YTHDC2 promoted cell proliferation in HCC, and its level was regulated by TNF-α-induced transcription factors c-Jun and ATF-2 [51].

In pancreatic ductal adenocarcinoma (PDAC), YTHDC2 was shown to recognize m6A modification sites on super-enhancer RNA (seRNA) and recruited H3K4 methyltransferase lysine methyltransferase 2A (MLL1), enhancing H3K4me3 modification, which facilitated chromatin accessibility of seRNAs and promoted oncogene transcription [52].

YTHDC2 was downregulated in colorectal cancer (CRC), leading to increased stability and expression of LIM domain kinase 1 (LIMK1) mRNA in CRC cells. LIMK1 overexpression promoted eIF2α phosphorylation, induced endoplasmic reticulum stress, and promoted stress granule formation, ultimately leading to resistance to 5-fluorouracil (5-FU) [53]. M6A methylation modification could influence the stability and degradation rate of microRNA (miRNA), thereby regulating tumor progression by modulating miRNA levels [54, 55]. YTHDC2 recognized m6A-modified miR-17-5p and promoted its degradation, resulting in increased expression of its downstream tumor suppressor gene Mitofusin 2 (MFN2), leading to decreased mitochondrial fusion and increased sensitivity to 5-FU in CRC [56]. However, another study revealed a significant positive correlation between YTHDC2 expression and CRC tumor staging. Mechanistically, YTHDC2 promoted the translation of transfer-related gene proteins under hypoxic conditions, such as Twist1 and hypoxia-inducible factor-1 alpha (HIF-1α), thereby facilitating CRC metastasis [57].

#### The role of YTHDC2 in endocrine system tumors

Papillary thyroid carcinoma (PTC) stands as the most prevalent form of endocrine malignancy [58]. YTHDC2 downregulated and exerted anti-cancer effects in both thyroid cancer tissues and cell lines. YTHDC2 curbed the proliferation of PTC cells and triggered their apoptosis by upregulating CYLD expression, leading to inactivation of the Akt pathway [59]. The METTL3-YTHDC2 axis has been indicated to maintain RNA stability of LINC00894 in an m6A-dependent manner, thereby inhibiting the lymphangiogenesis of vascular endothelial cells and the proliferation of PTC cells through the Hippo signaling pathway [60]. Moreover, the METTL16-YTHDC2 axis boosted the m6A modification of stearoyl-CoA desaturase-1 (SCD1) in PTC cells, accelerating its mRNA degradation and inhibit lipid metabolism, thereby inhibiting the growth of PTC cells [61].

#### The role of YTHDC2 in other systems tumors

YTHDC2 was also significantly upregulated in patients with prostate cancer (PC), especially in those with higher Gleason grading and serum prostate-specific antigen levels. Cell experiments have demonstrated that YTHDC2 overexpression notably boosted the growth, migration, and invasion capabilities of PC cells, suggesting a potential association between YTHDC2 upregulation and PC prognosis [62]. YTHDC2 has also been shown to be highly expressed in PC tissues and cells, regulated by circMID1/miR-330-3p. The overexpression of YTHDC2 activates the AKT signaling pathway by enhancing the expression of the oncogene IGF1R, thereby promoting the proliferation, migration, invasion, and glycolysis levels of PC cells [63]. Furthermore, YTHDC2 has been found to be highly expressed in radiation resistant nasopharyngeal carcinoma (NPC) cells, and YTHDC2 can bind to the mRNA of IGF1R and promote its expression. Mechanistically, overexpressed IGF1R promotes the resistance of NPC cells to radiotherapy by activating the IGF1R/ATK/S6 signaling pathway, which may become a potential target for radiosensitization therapy [64]. Compared to normal human skin, YTHDC2 was upregulated in human cutaneous squamous cell carcinoma (cSCC). YTHDC2 inhibited DNA damage repair induced by Ultraviolet B by inhibiting the expression of the tumor suppressor gene PTEN, suggesting its potential upregulation could serve as a biomarker for cSCC [65].

#### YTHDC2 as a tumor prognostic marker

YTHDC2 plays a pivotal role in cancer biology, exerting varied effects on tumor progression depending on its downstream targets. A comprehensive pan-cancer analysis underscored its significance [66], revealing impacts on epigenetic modifications and immune infiltration across diverse cancer types. Notably, mutations and methylation levels of YTHDC2 correlated with prognosis in specific cancers, with high diagnostic value demonstrated in cholangiocarcinoma, lung squamous cell carcinoma, thyroid carcinoma, ovarian serous cystadenocarcinoma, skin cutaneous melanoma (SKCM), testicular germ cell tumors, and uterine carcinosarcoma, among others. Additionally, it held prognostic significance for brain lower-grade glioma, rectum adenocarcinoma, and SKCM [64].

In head and neck squamous cell carcinoma, the low expression of YTHDC2 correlated with lower overall survival (OS), recurrence-free survival, and reduced immune infiltration levels, suggesting its potential as a prognostic and immune infiltration marker for head and neck squamous cell carcinoma [67]. Similarly, in rectal adenocarcinoma, decreased YTHDC2 expression was associated with significantly lower OS rates [68]. In NSCLC, low YTHDC2 expression correlated with a poorer prognosis, increased tumor malignancy, lymph node metastasis, larger tumor size, and advanced staging [69].

Additionally, genetic variations within the YTHDC2 promoter, such as the single nucleotide polymorphism (SNP) rs2416282, influence YTHDC2 expression and are associated with esophageal squamous cell carcinoma risk [70]. Another SNP within YTHDC2, rs7202116, shows a significant association with the survival of patients with HCC treated with transarterial chemoembolization, particularly with a poor prognosis observed in patients with the rs7202116 GG genotype. Additionally, the study highlights the regulatory influence of rs7202116 on FTO gene expression. These findings support the potential significance of m6A-regulated genes in HCC treatment strategies [71].

In CRC, conflicting findings exist regarding YTHDC2 expression and prognosis. While some studies using Kaplan Meier analysis reported reduced YTHDC2 expression associated with poor progression-free survival and OS [72], others indicated YTHDC2 upregulation linked to adverse prognosis, clinical features, and immune infiltration [73]. This inconsistency underscores the need for further investigation into YTHDC2’s role in CRC.

In PC, YTHDC2 was significantly upregulated, positively correlating with Gleason grading and being notably higher in lymph node metastasis castration-resistant prostate cancer (CRPC) compared to CRPC with bone metastasis [74]. While current research suggests YTHDC2 as a potential tumor biomarker aiding in diagnosis, prognostic evaluation, and treatment selection, further studies are essential to elucidate its mechanisms and clinical applications fully. With ongoing research, YTHDC2 holds promise for advancing personalized tumor treatment and precision medicine initiatives.

#### The role of YTHDC2 in non-tumor pathological processes

In addition to its research in tumors, the role of YTHDC2 in non-tumor diseases has also attracted the attention of researchers in recent years. Studies have shown that it may play an important role in reproductive system diseases, neurological diseases, immune system diseases, and metabolic diseases.The RNA targets of YTHDC2 and their roles in non-tumor pathophysiological processes are summarized in Table 1.

**Table 1:** The role of YTHDC2 in non-tumor pathological processes

Pathological processes	Tatget RNA	Effect on target RNA	Roles of target RNAs	References
Reproductive cell meiosis	CCNA2	Promote mRNA stability	Promote the transition from mitosis to meiosis	[33]
Spermatogenesis dysfunction	CCNB2	Promote mRNA stability	Reduce cell cycle arrest and improve reproductive toxicity	[81]
Neurogenesis	LRP2	Promote mRNA stability and translation efficiency	Facilitates neurogenesis and elicits antidepressant-like effects	[83]
Neural differentiation	HERV-H	Revent epigenetic silencing	Inhibit neural differentiation	[84]
Virus infection	IFN-β	Promote mRNA degradation	Promote innate immune response against viruses	[85]
KSHV infection	IL-6	Promote mRNA stability	Promote inflammatory response	[86]
TM infection	TLR2	Inhibit mRNA expression	Promote inflammatory response	[89]
Experimental autoimmune uveitis	ASH1L	Promote mRNA stability	Inhibits autoreactive Th17 cell responses in experimental autoimmune uveitis	[90]
Rheumatoid arthritis	AMIGO2	Promote mRNA expression	Promote the proliferation and aggressive behaviors of rheumatoid arthritis fibroblast-like synoviocytes	[94]
Diabetic peripheral neuropathy	KDM5B	Inhibit mRNA stability	Regulate mitochondrial metabolic reprogramming	[95]
VSMCs dysfunction	circYTHDC2	Promote RNA stability	Promote the proliferation and migration of VSMCs	[96]
Obesity	HIF1A	Promote mRNA translation efficiency	Promote the browning of white adipocytes and thermogenesis	[100]
Hepatic drug metabolism	CYP2C8	Promote mRNA degradation	Promote hepatic drug metabolism	[103]
Hepatic drug and lipid metabolism	CES2	Promote mRNA degradation	Promote hydrolysis of drugs and endogenous substrates	[107]
Osteogenic differentiation process	RUNX2	Promote mRNA degradation	Promote bone mesenchymal stem cells osteogenic differentiation	[110]
Inherited retinal dystrophies	PPEF2 and PDE6B	Promote mRNA translation efficiency	Regulate retinal function and inhibiting progressive rod death	[113]

This table summarizes the RNA targets of YTHDC2 and the role of its RNA targets in non-tumor pathophysiological processes

#### The role of YTHDC2 in the reproductive system

YTHDC2 exhibits tissue-specific expression in the human body. The highest levels have been observed in the testes, underscoring their crucial role in regulating the development, maturation, and function of germ cells [21]. It has been shown to govern prophase I of meiosis in mammals, safeguarding against telomere aggregation by preserving the meiotic transcriptome and preventing microtubule network alterations [75]. In mice, prolonged meiotic prophase I maintenance requires the Meioc gene, a conserved germ cell-specific factor across most metazoans [76]. YTHDC2 collaborated with MEIOC to drive the meiotic cell cycle program via post-transcriptional control of target transcripts [77].

Deficiencies in YTHDC2/MEIOC could trigger premature entry into aberrant stages and germ cell apoptosis, disrupting the transition from spermatogonia to meiotic gene expression programs [78]. The RBM46/YTHDC2/MEIOC complex, comprising YTHDC2 and its partner MEIOC, stood as a principal post-transcriptional regulator governing mitotic diversion and mitigating mitotic transcript levels during mammalian spermatogenesis [79]. YTHDC2 facilitated the transition from mitosis to meiosis in mammalian germ cells, regulating germ cell progression during meiosis by maintaining post-transcriptional cyclin A2 (CCNA2) RNA levels, pivotal in determining cell fate [33].

Studies by Hsu et al. revealed that YTHDC2 knockout in mouse testes impaired translation efficiency and mRNA abundance of its targets, impeding germ cell transition from mitosis to meiosis. Consequently, YTHDC2 gene knockout mice exhibited significantly smaller testes and ovaries, alongside infertility [21]. Notably, the m6A binding pocket mutation in YTHDC2 minimally impacted germ cell development and mouse fertility, indicating YTHDC2’s function was m6A-independent [30]. Li et al. demonstrated that mice harboring YTH point mutations within YTHDC2 remained capable of reproduction, yet loss of their 3’ → 5’ RNA helicase activity rendered them infertile. Additionally, exonuclease XRN1 enhanced YTHDC2’s helicase activity, sustaining its function [30].

YTHDC2’s role extends to female germ cells (FGCs), crucial for meiosis initiation and progression. YTHDC2 augmented TRA8-positive FGCs and significantly altered FGC distribution during zygotene and pachytene stages [80]. Manganese exerted toxic effects on male reproduction, with occupational exposure linked to decreased semen quality in male workers. Mechanistically, Manganese inhibited the YTHDC2/CCNB2 signaling pathway, stalling the G2/M cell cycle phase. Elevated YTHDC2 expression enhanced CCNB2 levels, ameliorating cell cycle arrest and mitigating reproductive toxicity post-Manganese exposure [81]. Furthermore, YTHDC2 expression in developing human embryonic ovaries and its upregulation in meiotic germ cells suggested its pivotal role in human meiosis. Various YTHDC2 variants, including pathogenic ones, were associated with primary ovarian insufficiency (POI), highlighting its significance in human meiosis regulation [82].

In summary, YTHDC2 emerges as a critical regulator in the human reproductive system, influencing germ cell development, function, and physiology through gene expression regulation and RNA processing mechanisms. Its role holds substantial implications for maintaining normal reproductive system function.

#### The role of YTHDC2 in the nervous system

Xu et al. found that reducing the expression of METTL3 or METTL 14 in neural stem cells (NSCs) significantly reduces the abundance of m6A, cell proliferation, and neuronal generation, while enhancing the differentiation of glial cells. Meanwhile, YTHDC2 promotes neuronal generation by promoting the stability and translation efficiency of m6A modified Lrp2 mRNA, which may reverse spatial memory decline and depressive like behavior, making it a promising antidepressant strategy [83]. YTHDC2 interacts with m6A modified HERV-H RNA, binds to the genomic site of LTR7/HERV-H, and recruits the DNA 5mC demethylase TET1 to prevent epigenetic silencing of LTR7/HERV-H. In human embryonic stem cells, the interaction between YTHDC2 and LTR7 inhibits neural differentiation [84].

#### The role of YTHDC2 in the immune system

YTHDC2 plays a crucial role in terminating the innate immune response at the late stage of infection to prevent unwanted inflammation. YTHDC2 degrades m6A modified IFN-β mRNA in the later stage of viral infection inhibits the innate immune response against viruses, thereby preventing unnecessary inflammation and maintaining the body's homeostasis [85]. The m6A modification plays an important role during viral infection, as the overall level of host methylation decreases during Kaposi's sarcoma associated herpesvirus (KSHV) infection. YTHDC2 helps IL-6 resist degradation caused by the viral endonuclease SOX during KSHV infection by recognizing the m6A modification site of 3'UTR on IL-6 mRNA, thereby playing an important role in the process of KSHV infection in cells [86].

The immune evasion of talaromyces marneffei (TM) is an important factor contributing to the high mortality rate of Marneffei’s candidiasis [87, 88]. Zhu et al. found that there were dynamic changes in overall m6A levels and upregulation of YTHDC2 expression in macrophages infected with TM. Knocking down YTHDC2 in TM infected cells showed a m6A dependent increase in TLR2 expression, leading to upregulation of inflammatory factors TNF-α and IL1-β [89].

The expression of METTL3 and m6A levels were significantly reduced in the eyeballs and T cells of experimental autoimmune uveitis (EAU). YTHDC2 enhances the mRNA stability of ASH1L in an m6A dependent manner to promote its expression, thereby inhibiting the expression of IL-17 and IL-23 receptors and reducing the response of pathogenic Th17 cells. This inhibits pathogenic Th17 cell responses both in vivo and in vitro, helping to alleviate the development of EAU [90].

The translation of the genome through the internal ribosome entry site (IRES) dependent mechanism is crucial in the process of hepatitis C virus (HCV) infection [91–93]. YTHDC2 can recognize m6A methylated adenosine at position nt 331 in the HCV RNA gene and support HCV IRES dependent translation under the synergistic effect of cellular La antigen. YTHDC2 plays an important role in the translation initiation dependent on HCV IRES, providing a potential novel therapeutic pathway for intervening in the process of HCV infection [27]. YTHDC2 and METTL3 jointly regulate the expression of adhesion molecule with Ig like domain 2 (AMIGO2), regulate the proliferation and invasion ability of rheumatoid arthritis synovial fibroblasts, and thereby affect the disease progression of rheumatoid arthritis [94].

#### The role of YTHDC2 in metabolic diseases

YTHDC2 plays a pivotal role in combating diabetic peripheral neuropathy (DPN) by enhancing mitochondrial metabolism. It does so by reducing KDM5B mRNA stability, thereby increasing SIRT3 expression, which in turn improves mitochondrial function, suggests YTHDC2 as a promising target for DPN treatment through mitochondrial metabolic reprogramming [95]. The expression of circYTHDC2 increases under high glucose conditions, and the knockout of circYTHDC2 significantly inhibits the proliferation and migration of vascular smooth muscle cells (VSMCs). YTHDC2 regulates the stability of circYTHDC2 through m6A modification, while circYTHDC2 negatively regulates the expression of TET2 by targeting the 3'UTR unstable motif of TET2, promoting the differentiation of VSMCs into synthetic ones [96]. These findings indicate that the YTHDC2/circYTHDC2/TET2 pathway is an important target for metformin to prevent the progression of high glucose induced VSMCs dysfunction. Zhou et al. found that the expression of YTHDC2 was significantly reduced in the liver of obese mice and non-alcoholic fatty liver disease (NAFLD) patients, and was associated with liver fat accumulation. Mechanistically, YTHDC2 can bind to the mRNA of lipid synthesis genes, thereby reducing their mRNA stability and inhibiting their gene expression [97]. This indicates that YTHDC2 also plays an important role in regulating liver lipid synthesis and triglyceride homeostasis, which has potential implications for the treatment of NAFLD associated with obesity. HIF1A is a transcription factor that can promote the browning process of adipocytes by activating the transcription of key thermogenic genes, and has potential therapeutic significance in combating obesity and metabolic diseases [98, 99]. The absence of FTO in adipose tissue leads to an increase in the m6A modification level of HIF1A mRNA, while YTHDC2 can promote the translation of HIF1A and increase the abundance of HIF1A protein in a m6A methylation dependent manner [100].

#### The role of YTHDC2 in other pathophysiological processes

CYP2C8 is a cytochrome P450 enzyme that plays an important role in drug metabolism [101, 102]. It mainly participates in the oxidative metabolism reaction of drugs, converting some drugs into more easily excreted metabolites, thereby affecting the metabolism and efficacy of drugs. CYP2C8 can be methylated with m6A by METTL3/14 in the liver and removed by FTO, while YTHDC2 recognizes m6A modified CYP2C8 mRNA and promotes its degradation, thereby regulating CYP2C8 expression and affecting drug metabolism [103]. Carboxyesterase 2 (CES2) is a serine esterase responsible for the hydrolysis of drugs and endogenous substrates such as triglycerides and diglycerides [104–106]. YTHDC2 downregulates the expression of CES2 by recognizing m6A in the 5 ‘untranslated region (UTR) of CES2, degrading mRNA containing m6A [107].

RUNX family transcription factor 2 (RUNX2) is a transcription factor that is one of the main regulatory factors for osteoblast differentiation, and has a significant impact on bone formation and the differentiation of bone marrow mesenchymal stem cells (BMSCs) into osteoblasts [108, 109]. During the osteogenic differentiation process of BMSCs, the expression of YTHDC2 protein decreases and leads to an increase in intracellular RUNX2 (mRNA and protein) expression levels, indicating that YTHDC2 is a promising molecular target for regulating the osteogenic differentiation of BMSCs [110]. Inherited retinal dystrophies are the leading cause of visual impairment and irreversible blindness worldwide, but their exact molecular and genetic mechanisms remain elusive [111, 112]. The deficiency of YTHDC2 in rod cells leads to impaired translation efficiency of PPEF2 and PDE6B, ultimately resulting in a decrease in protein levels in the retina, leading to gradual cell death and impaired retinal function [113].

## Conclusion and prospects

YTHDC2, serving as a critical RNA binding protein, plays a fundamental role in governing biological processes such as RNA translation and stability. The expression changes of its target genes are related to the progression of many diseases, including cancer and non-tumor pathological processes. Extensive research has established a close correlation between YTHDC2 and various diseases, indicating its potential as a therapeutic target in these diseases. In this review, we introduced the structure and function of YTHDC2, with a focus on summarizing its roles and regulatory mechanisms in cancer and other physiological and pathological processes. Looking forward, delving deeper into the specific mechanisms of YTHDC2 in RNA regulation and investigating its involvement in the gene regulatory network through more expansive research will be paramount. This undertaking will substantially contribute to unraveling the mechanisms underpinning these diseases and identifying potential treatment approaches.

Recent years have seen significant advancements in comprehending YTHDC2, yielding crucial insights into its functionality and mechanism of action. These discoveries also open up new avenues and possibilities for developing therapeutic drugs aimed at targeting YTHDC2. Some small molecule compounds have been found to interfere with the activity of m6A readers, thereby affecting their regulation of RNA function. For example, Tegaserod, a 5-HT4 receptor agonist, has been found to block the direct binding of YTHDF1 to m6A-modified mRNA. In acute myeloid leukemia (AML), Tegaserod can inhibit the translation of cyclin E2 regulated by YTHDF1, thereby affecting the proliferation and survival abilities of AML cells [114]. BTYNB is a small molecule compound found to have an inhibitory effect on IGF2BP1. BTYNB can selectively inhibit the binding of IGF2BP1 to its targets, thereby reducing the expression levels of its target mRNA and protein. BTYNB can effectively inhibit the proliferation of IGF2BP1-positive ovarian cancer and melanoma cells, while having no effect on IGF2BP1-negative cells [115]. CWI1-2 is a newly discovered small molecule compound found to inhibit IGF2BP2. Research has shown that by inhibiting IGF2BP2, CWI1-2 can regulate the expression of key targets (such as MYC, GPT2, and SLC1A5) in the glutamine metabolism pathway in an m6A-dependent manner, thereby inhibiting the development of AML and the self-renewal of leukemia stem cells [116]. JX5 is a small molecule compound with an inhibitory effect on IGF2BP2. JX5 inhibits the binding of IGF2BP2 to the oncogene NOTCH1 in T-ALL, thereby directly suppressing the proliferation of T-ALL in an m6A-dependent manner. Treatment with JX5 in T-ALL can produce effects similar to knocking out IGF2BP2, inhibiting the proliferation of T-ALL cells and prolonging animal survival [117]. These studies indicate that m6A reader inhibitors can effectively inhibit tumor progression, providing new potential treatment strategies for cancer patients. Currently, these new m6A reader drugs still face some challenges in clinical application, including issues such as drug bioavailability, toxicity, and side effects. Additionally, since m6A reader proteins also play important roles in normal cellular biological processes, inhibitors may have adverse effects on normal cell functions, leading to off-target effects and toxic reactions.

As of now, there have been no detailed research reports on small molecule inhibitors targeting YTHDC2. With further research on the mechanisms of action of YTHDC2 in cancer, there may be more studies on small molecule inhibitors targeting YTHDC2 in the future. These potential small molecule inhibitors may help explore the role of YTHDC2 in cancer development and provide new insights for the development of treatment strategies targeting YTHDC2.

## Abbreviations

m6A: N6-methyladenosine
MTC: M6A methyltransferase complex
METTL3: Methyltransferase like 3
METTL14: Methyltransferase like 14
WTAP: Wilms tumor 1-associated protein
RBM15: RNA-binding motif protein 15
SAM: S-adenosylmethionine
FTO: Fat mass and obesity-related protein
ALKBH5: Alkylation repair homolog protein 5
YTH: YT521-B homology
CLIP: Crosslinking immunoprecipitation
CDS: Coding region
SOX9: Sex determining region Y-box 9
LUAD: Lung adenocarcinoma
CYLD: Cylindromatosis
SLC7A11: Solute carrier 7A11
UTR: Untranslated region
HOXA13: Homeobox A13
SLC3A2: Solid carrier 3A2
MRPL12: Mitochondrial ribosomal protein L12
NSCLC: Non-small cell lung cancer
ID3: Inhibitor of DNA-binding 3
lncRNAs: Long non-coding RNAs
BTNL9: Butyrophilin like 9
eIF4GI: Eukaryotic translation initiation factor 4 gamma 1
VEGFA: Vascular endothelial growth factor A
GC: Gastric cancer
YAP: Yes1 associated transcriptional regulator
HCC: Hepatocellular carcinoma
ATG5: Autophagy related 5
PDAC: Pancreatic ductal adenocarcinoma
MLL1: Lysine methyltransferase 2A
CRC: Colorectal cancer
LIMK1: LIM domain kinase 1
5-FU: 5-Fluorouracil
miRNA: MicroRNA
MFN2: Mitofusin 2
HIF-1α: Hypoxia-inducible factor-1 alpha
PTC: Papillary thyroid carcinoma
PC: Prostate cancer
NPC: Nasopharyngeal carcinoma
cSCC: Cutaneous squamous cell carcinoma
SKCM: Skin cutaneous melanoma
OS: Overall survival
SNP: Nucleotide polymorphism
CRPC: Castration-resistant prostate cancer
CCNA2: Cyclin A2
FGCs: Female germ cells
NSCs: Neural stem cells
KSHV: Kaposi’s sarcoma associated herpesvirus
TM: Talaromyces marneffei
EAU: Experimental autoimmune uveitis
HCV: Hepatitis C virus
AMIGO2: Adhesion molecule with Ig like domain 2
DPN: Diabetic peripheral neuropathy
VSMCs: Vascular smooth muscle cells
NAFLD: Non-alcoholic fatty liver disease
CES2: Carboxyesterase 2
RUNX2: RUNX family transcription factor 2
BMSCs: Bone marrow mesenchymal stem cells
AML: Acute myeloid leukemia
T-ALL: T-cell acute lymphoblastic leukemia
